# The impact of involuntary-to-voluntary hospitalization transitions on psychiatric decompensation: A protocol for a retrospective cohort study

**DOI:** 10.1192/j.eurpsy.2025.2185

**Published:** 2025-08-26

**Authors:** M. Castro, J. Coelho, A. R. Ferreira, H. Salgado

**Affiliations:** 1 Faculty of Medicine, University of Porto; 2 Psychiatry Service, Unidade Local de Saúde São João; 3 Department of Clinical Neurosciences and Mental Health, Faculty of Medicine, University of Porto; 4 CINTESIS@RISE, Faculty of Medicine, University of Porto, Porto, Portugal

## Abstract

**Introduction:**

Involuntary hospitalization of a patient with a mental disorder is defined as admission to an inpatient unit without consent. Literature suggests that severe involuntarily admitted patients often present in crisis situations, receive less pharmacologic intervention and therapy, and exhibit poorer prognostic outcomes, such as an increased risk of readmission. Despite being a lifesaving treatment, involuntary admissions can also be stigmatizing, undermine the long-term therapeutic relationship and reduce adherence to care. In this context, little research has been conducted to evaluate how switching a patient’s hospitalization from involuntary to voluntary may impact health outcomes, such as psychiatric decompensation.

**Objectives:**

To compare the risk of hospital readmission of patients who switched to voluntary hospitalization with those who remain under involuntary hospitalization, and to analyze their sociodemographic characteristics and prognostic outcomes.

**Methods:**

An observational retrospective study will be conducted using administrative and clinical data of patients who were involuntary admitted to inpatient psychiatry of Unidade Local de Saúde São João. All involuntary hospitalizations spanning from January 1, 2022, and December 31, 2022, will be categorized into two groups: patients who switch to voluntary hospitalization or patients that maintained involuntary hospitalization. Data registered in medical records within one year after the index hospitalization will be assessed (whether structured data or free-text). Descriptive, univariate, and multivariate analyses will be performed.

**Results:**

For both groups, sociodemographic and clinical variables will be described and compared, as well as the number of previous admissions, their legal status and the presence of previous ambulatory involuntary treatment. Administrative data on patient’s hospitalization, such as the length of stay, medical treatment and procedures performed, and the orientation received after discharge will also be compared. Additionally, prognosis outcomes, including readmissions, length of stay of readmissions and legal status of readmission will be analyzed.

**Conclusions:**

We expect to elucidate the impact of switching involuntary hospitalized patients to voluntary status on prognosis outcomes. Through this comparative analysis, we hope to provide evidence supporting the prioritization of voluntary treatment whenever feasible.

**Disclosure of Interest:**

None Declared

